# Establishment of fingerprint of phenolic compounds in *Semen Ziziphi Spinosae* and study on the spectrum-effect relationship based on different preceding cropping areas

**DOI:** 10.3389/fchem.2024.1520586

**Published:** 2025-01-03

**Authors:** Junfeng Jiang, Jun Luo, Wenyu Zheng, Jiayi Liu, Hui Jiang, Cuiyun Wu, Hongjin Bai

**Affiliations:** ^1^ College of Chemistry and Chemical Engineering, Tarim University, Alar, Xinjiang, China; ^2^ College of Horticulture and Forestry, Tarim University, Alar, Xinjiang, China

**Keywords:** *Semen Ziziphi Spinosae*, phenolic compounds, preceding cropping area, antioxidant activity, fingerprint, spectrum-effect relationship

## Abstract

As an agricultural planting practice, preceding cropping can not only enhance soil fertility and reduce pests and diseases but also boost crop yield and quality. In this study, SZS samples from different preceding cropping areas were selected as research subjects. Phenolic compounds were analyzed using high-performance liquid chromatography (HPLC), and antioxidant activities were assessed based on free radical scavenging effects. Variety differences were explored through chemical pattern recognition, and the spectrum-effect relationship between the fingerprint spectra of SZS and antioxidant activity was investigated using Pearson correlation analysis, grey relational analysis, and other methods. A total of 17 peaks were observed, among which 4 peaks were identified. They are gallic acid, catechin, spinosin, and scutellarin. The 22 SZS samples could be categorized into 3 groups, with cluster analysis and principal component analysis results being largely consistent. Spinosin, a marker compound of SZS, is a crucial contributor to the total antioxidant activity. In conclusion, the spectrum-effect relationship between phenolic compounds and the antioxidant activity of SZS was established, and the main characteristic components affecting antioxidant activity were identified, providing a reference for the quality evaluation of SZS and the development of its products.

## 1 Introduction

*Semen Ziziphi Spinosae* (SZS) is one of the first batch of traditional Chinese medicinal materials recognized by the Ministry of Health of China as both food and medicine. SZS has a long history of medicinal and edible use, and its application value is significant, being widely used in traditional Chinese patent medicines and health products. Historically, it has been recorded that SZS “can be used for treating insomnia, thirst, and night sweats when ripe; when raw, they can induce sound sleep and are considered medicines for the liver and gallbladder.” Studies have shown that there are many compounds in SZS, mainly including phenolic acids, terpenes, flavonoids, and fatty acids (Fan et al., 2022; Wei and Lin, 2022), which have unique effects in anti-depression (Van Straten et al., 2018), insomnia (Bai et al., 2018), antioxidant (Dias et al., 2021; Havsteen, 2002), blood pressure reduction (Gu et al., 2019), and memory improvement (Zhang et al., 2020), and are widely used in clinical applications (Wang et al., 2022). Phenolic compounds, as one of the main active components of SZS (Vinson et al., 2001; Yan et al., 2019), also have rich nutritional value and excellent antioxidant activity. They are a natural green antioxidant and play an indispensable role in the effects of SZS (Shang et al., 2022).

Long-term monoculture of crops often leads to changes in soil physical and chemical properties, soil autotoxicity, and microbial community, which is unfavorable for plant growth and yield, resulting in continuous cropping obstacles (Liu et al., 2015; Bennett et al., 2012). Some studies have shown that crop rotation can improve soil enzyme activity, reduce soil autotoxicity, and affect the diversity and abundance of soil microbial communities, thereby affecting crop growth and yield (Koch et al., 2018; He et al., 2019). Accordingly, we selected three different areas where the preceding crops were almond, carrot, and cotton to plant SZS. Through the investigation of the phenolic compounds in SZS, the quality evaluation standard of SZS can be constructed relatively quickly.

The HPLC fingerprint spectrum, as an important analytical method in the modern scientific field, can effectively reflect the main chemical components and characteristic information of traditional Chinese medicines to a certain extent (Yang et al., 2024). In this study, based on high-performance liquid chromatography and relying on the pharmacospectroscopy of traditional Chinese medicines, the fingerprint spectra of phenolic compounds in SZS from different areas were constructed to investigate the differences of phenolic compounds in SZS from different areas (Zhang et al., 2024). Taking the free radical scavenging abilities of DPPH and ABTS as indicators, the spectrum-effect relationship between the HPLC fingerprint spectra of phenolic compounds in SZS and the antioxidant activity was established (Tena et al., 2020). Combined with Pearson correlation analysis, grey relational analysis, and partial least squares method, the correlations between the common peaks and the antioxidant activity were analyzed, and the main antioxidant active components were screened out to provide references for the practical application and research and development of SZS later.

## 2 Materials and methods

### 2.1 Materials and reagents

The principle of random sampling was adopted to randomly sample SZS from different areas in proportion, and a total of 22 SZS samples were obtained. Sample S1 and S2 are planted in almond fields, S3∼S17 are planted in carrot fields, and S18∼S22 are planted in cotton fields. Glacial acetic acid (CH_3_COOH, ≥99.5%), potassium persulfate (K_2_O_8_S_2_, ≥99.0%), 2,2′-Azino-bis(3-ethylbenzothiazoline-6-sulfonic acid) (ABTS, ≥99%), 2,2-Diphenyl-1-picrylhydrazyl (DPPH, ≥99%)and HPLC-grade methanol were purchased from Shanghai Macklin Company; reference substances: Gallic acid, Catechin, Spinosin, Scutellarin were purchased from Shanghai Yuanye Biotechnology Co., Ltd., and the quality of all reference substances was determined to be ≥ 98%.

### 2.2 Preparation of the test sample solution

The collected SZS was crushed using a multifunctional food processor and ground into a powder, which was then sealed and stored at −20°C for later use. Following the method described in (Zhao et al., 2019) with suitable modifications, the SZS was degreased. Initially, 1 g of SZS powder was passed through a 40-mesh (0.38 mm) sieve, followed by the addition of 10 mL of petroleum ether. The degreasing process was conducted with ultrasonic assistance at 80 W and 25°C for 30 min. After allowing the mixture to stand for 1 h, it was centrifuged at 10,000 r/min at room temperature for 10 min. The supernatant was discarded, and the degreased powder was placed in a fume hood to evaporate any residual petroleum ether, yielding the degreased SZS powder. Subsequently, 0.5 g of the degreased SZS powder was extracted with an 80% methanol solution using ultrasonication at 20°C and 200 W for 30 min. After centrifugation, the supernatant was collected, rotary evaporated and concentrated, then dissolved in methanol to prepare the test sample solution (Iftikhar et al., 2020).

### 2.3 Preparation of the reference solution

Accurate amounts of gallic acid, catechin, spinosin, and scutellarin reference substances were weighed and dissolved in methanol to prepare a mixed reference solution with a mass concentration of 1 mg/mL. The solution was thoroughly mixed, filtered, and stored at −20°C for later use. For use, the solution was diluted with methanol to achieve a reference solution with a mass concentration of 0.1 mg/mL, filtered through a 0.22 μm membrane, and then injected following the chromatographic conditions described in section “2.4”.

### 2.4 Chromatographic conditions

Chromatographic column: Cosmosil C18 (4.6 mm × 250 mm, 5 μm). Mobile phase: Methanol (A)-0.1% acetic acid in water solution (B); Gradient elution program: 0–8 min, 5%–15% A; 8–25 min, 15%–35% A; 25–45 min, 35%–60% A; 45–55 min, 60% A; 55–60 min, 60%–85% A; 60–70 min, 85% A. Flow rate: 1.0 mL/min, injection volume: 10 μL, column temperature: 35°C, and detection wavelength: 275 nm.

### 2.5 Determination of total phenolic content

Employing the method described by Siano et al., 2019 with suitable modifications, the SZS sample solution was prepared as described in section “2.2”. To different test tubes, 0.4 mL of the sample solution was added, followed by 10 mL of water and 0.3 mL of 1 N Folin-Ciocalteu reagent. After thorough mixing and shaking, the reaction was allowed to proceed at room temperature in the dark for 5 min. Subsequently, 1 mL of 15% Na_2_CO_3_ solution was added, mixed and shaken well, and the reaction was allowed to proceed at room temperature in the dark for 2 h. Absorbance was measured at 751 nm to obtain the sample absorbance data, which were calculated based on gallic acid, with triplicate determinations performed.

### 2.6 DPPH free radical scavenging experiment

The DPPH free radical scavenging rate of phenolic compounds in SZS was determined following the method described by Tian et al. (2016) with appropriate modifications. SZS sample solutions with concentrations of 0.2, 0.5, 1, 2, and 4 mg/mL were prepared sequentially as described in section “2.2”. A 0.1 mmol/L DPPH solution was prepared. The extracts of the SZS samples were diluted 5-fold. 100 μL of the diluted SZS extracts were mixed with 150 μL of DPPH solution and the reaction was allowed to proceed at room temperature in the dark for 30 min. Absorbance was measured using a microplate reader at 517 nm and recorded as A1; the control group absorbance as A2; and the blank group absorbance as A0. All experiments were performed in triplicate. The DPPH scavenging rate was calculated by using Equation 1.

$$DPPH\;scavenging=\left(1-\frac{A1-A2}{A0}\right)\times 100\%$$ (1)

### 2.7 ABTS free radical scavenging experiment

The ABTS free radical scavenging ability was assessed following the method described by Fang et al. (2009) with appropriate modifications. The sample solutions were prepared as described in section “2.2”. A 7 mmol/L ABTS aqueous solution and a 2.45 mmol/L potassium persulfate solution were prepared. The ABTS solution and potassium persulfate solution were mixed in equal volumes and stored at 4°C for 18 h. For use, an appropriate amount was taken and diluted 50-fold. The SZS sample solutions were diluted 25-fold. 100 μL of the diluted SZS sample solutions were mixed with 150 μL of ABTS solution, shaken well, and the reaction was allowed to proceed in the dark for 6 min. Absorbance was measured using a microplate reader at 734 nm and recorded as A1; the control group absorbance as A2; and the blank group absorbance as A0. All experiments were performed in triplicate. The ABTS scavenging rate was calculated by using Equation 2.

$$ABTS\;scavenging=\left(1-\frac{A1-A2}{A0}\right)\times 100\%$$ (2)

### 2.8 Fingerprint methodological examination

#### 2.8.1 Precision test

An appropriate amount of the test sample solution was injected six times consecutively under the conditions described in section “2.4”to calculate the RSD values for the relative retention time and relative peak area of the 17 common peaks. If the RSD of the relative retention time for each common peak is less than 0.5%, and the RSD of the relative peak area is less than 2.5%, this indicates good precision of the instrument.

#### 2.8.2 Stability test

The same batch of test sample solution was injected at 0, 4, 8, 12, 24, and 48 h under the chromatographic conditions specified in section “2.4”, and the relative retention time and peak area for each common peak were recorded. If the RSD of the relative retention time for each common peak is less than 0.1%, and the calculated RSD of the relative peak area is less than 3.0%, this indicates that the test sample solution remains stable within 48 h.

#### 2.8.3 Reproducibility test

An appropriate amount of the same test sample solution was injected in six batches according to the chromatographic conditions specified in section “2.4”to calculate the relative retention time and peak area for each common peak. The results indicate that if the RSD of the relative retention time for each common peak is less than 0.6%, and the RSD of the relative peak area is less than 1.7%, this demonstrates good reproducibility of the method.

## 3 Results and discussion

### 3.1 Establishment of fingerprint

**FIGURE 1:**
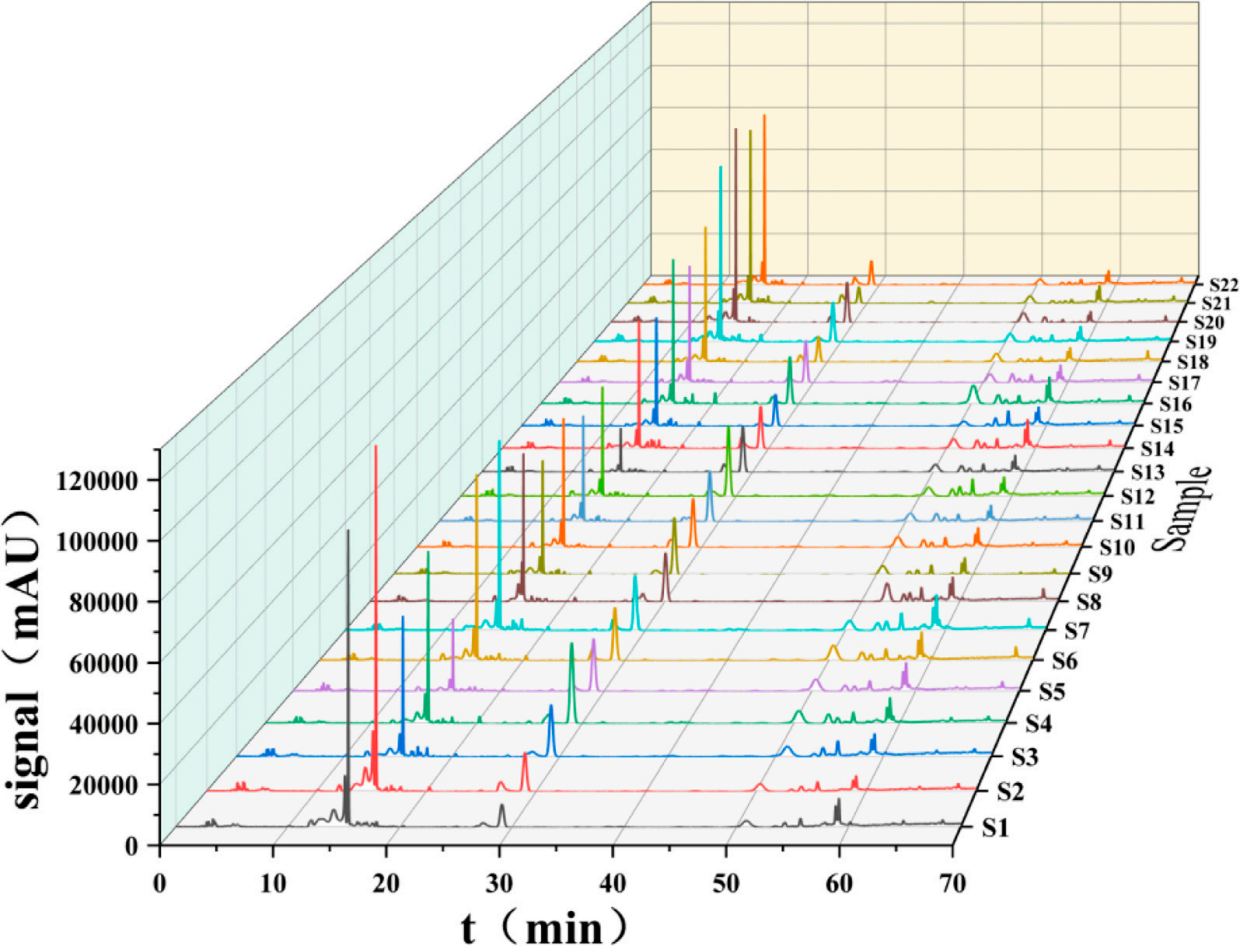
SZS fingerprints of 22 samples.

**FIGURE 2:**
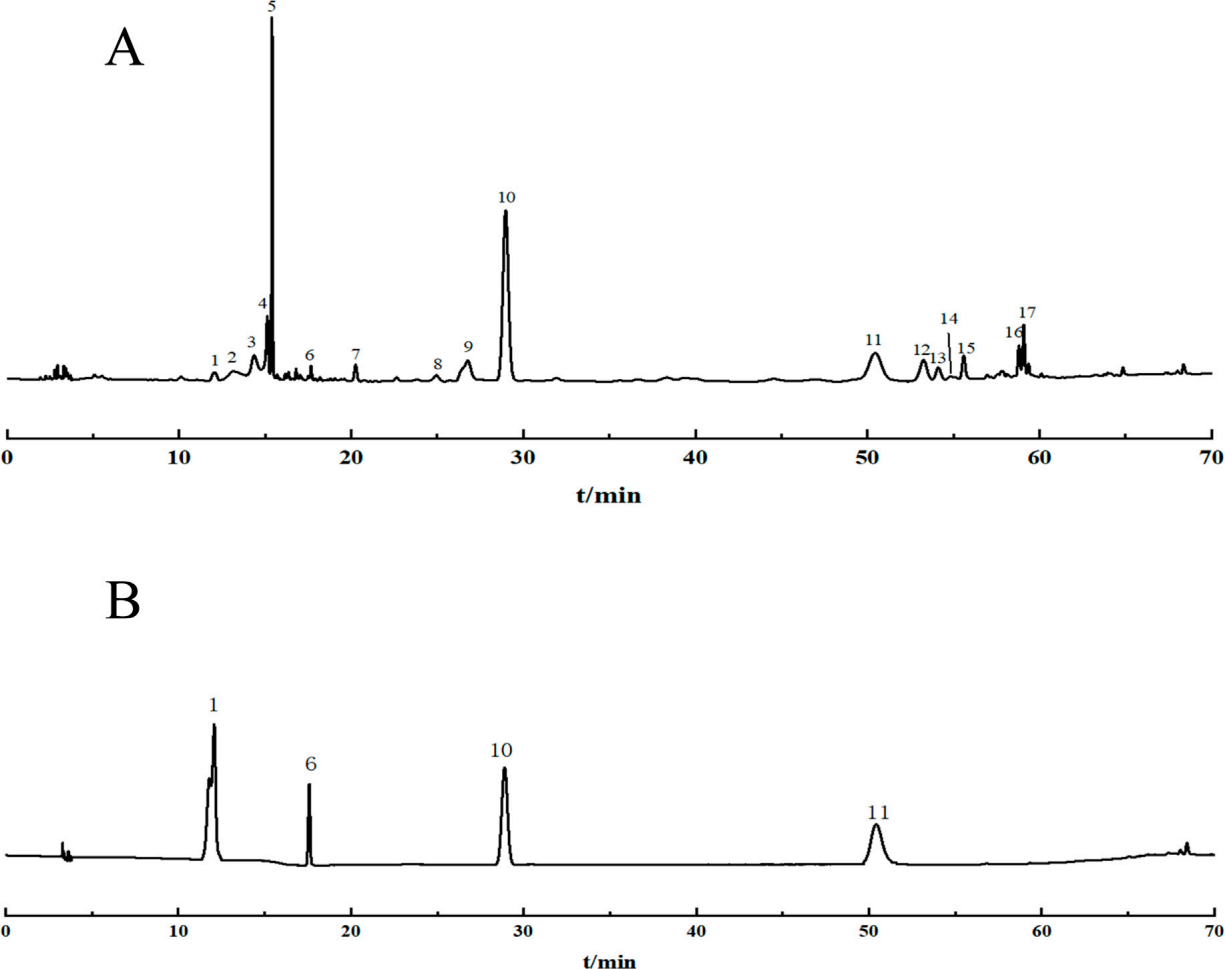
**(A)** Chromatogram of SZS samples; **(B)** Chromatogram of mixed reference substance.

The 22 prepared test sample solutions and the reference solution were injected under the chromatographic conditions described in section “2.4”to generate the superimposed HPLC fingerprint spectra (Figure 1). For instance, in sample S4, a total of 17 common peaks were identified, and 4 compounds were characterized as gallic acid (X1), catechin (X6), spinosin (X10), and scutellarin (X11), with spinosin being the marker compound for SZS. Chromatograms are presented in Figures 2A, B.

### 3.2 Determination results of phenolic compound content

The mixed reference solution was accurately aspirated and diluted with methanol to create a series of dilutions. Solutions were injected and analyzed under the chromatographic conditions described in section “2.4”to generate the reference standard curve and perform regression analysis. As shown in Supplementary Table S1, all R2 values exceeded 0.9997, indicating a strong linear relationship. The phenolic compound contents in the 22 SZS samples are detailed in Supplementary Table S2.

### 3.3 Similarity analysis

Similarity evaluation can effectively ensure the quality stability of traditional Chinese medicines, mitigate additional risks due to quality fluctuations, and control subsequent processing and production to ensure product quality. Additionally, similarity evaluation is a crucial method for verifying the authenticity of traditional Chinese medicines (Xiao et al., 2023). The liquid chromatographic similarities among the 22 SZS samples ranged from 0.832 to 0.993 (Supplementary Table S2), indicating good similarity across different SZS sample.

### 3.4 Cluster analysis

**FIGURE 3:**
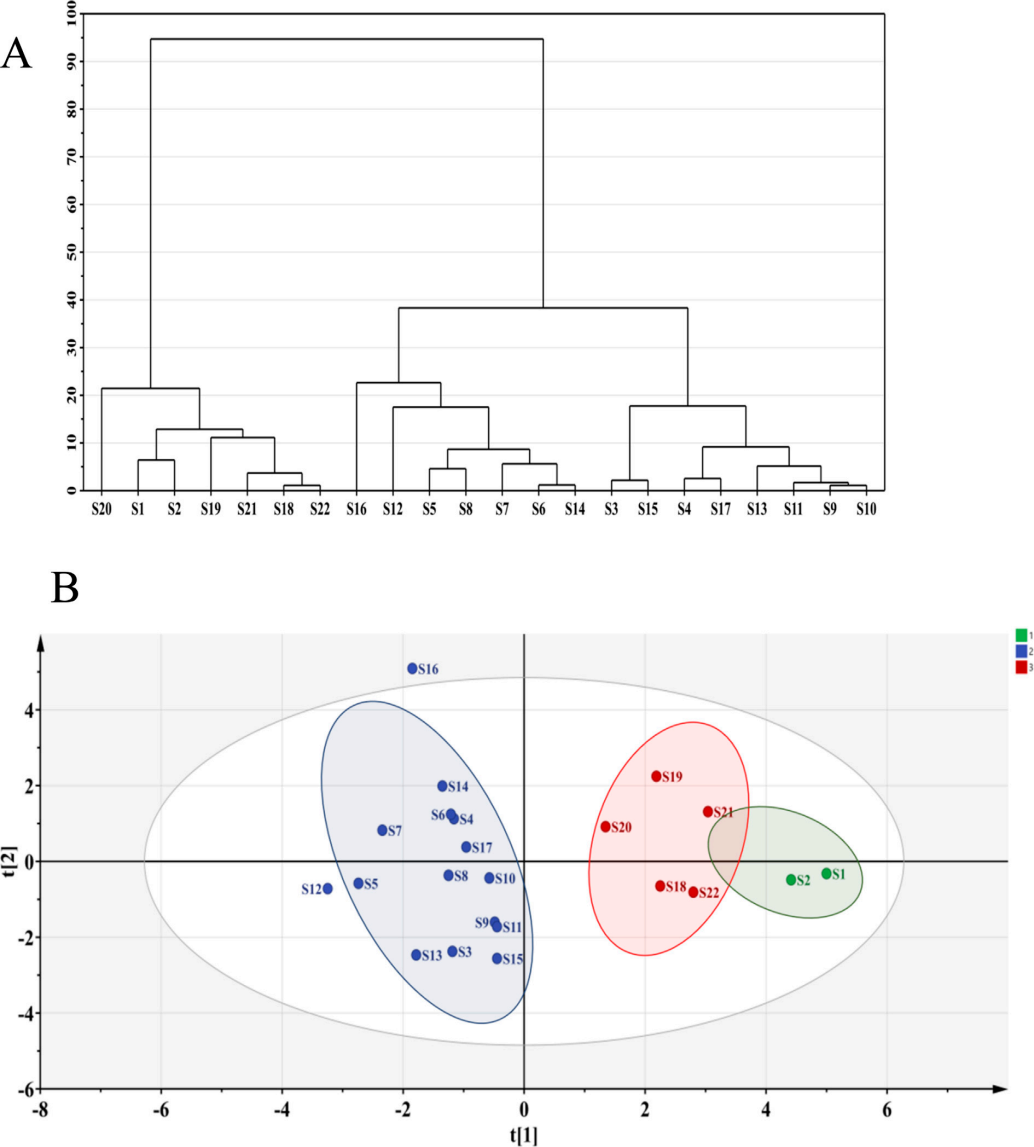
**(A)** Cluster analysis of 22 SZS samples; **(B)** Principal component analysis.

High-performance liquid chromatography data from different SZS sample were imported into SPSS 27.0 software for analysis. Using the peak areas of the common peaks as variables, cluster analysis was performed using the between-group linkage method and squared Euclidean distance as the measurement standard (Zhao et al., 2023). As depicted in Figure 3A, the 22 SZS samples could be categorized into two major groups. Samples S1 and S2, originating from the almond field, formed a subgroup, while S18-S22, from the cotton field, showed a certain correlation, thus being grouped into a larger category; the remaining samples formed another group, all originating from the carrot field. The cluster analysis revealed distinct regional characteristics across different categories, indicating a stronger correlation among samples from the same area. Hence, they were clustered together.

### 3.5 Principal component analysis

Principal component analysis, a widely used method for dimensionality reduction and multivariate analysis (Szymańska-Chargot et al., 2024), effectively manages correlations between multiple variables and identifies representative principal components. By using principal components to represent other variables, it minimizes distortion, effectively reducing dimensionality while accurately representing the data. The peak areas of the common peaks from the 22 SZS samples were used as variables and imported into SIMCA 14.1 software for principal component analysis. Results are presented in Figure 3B. The SZS samples were categorized into three groups. Specifically, S1 and S2 formed one group, both originating from the almond field; S3 to S17 formed another group, all from the carrot field; S19 to S22 formed the third group, all planted in the cotton field. Notably, S1, S2, and S19-S22 could also be grouped into a larger category, indicating a close relationship among them. These results were largely consistent with the cluster analysis findings.

### 3.6 Antioxidant activity examination

**FIGURE 4:**
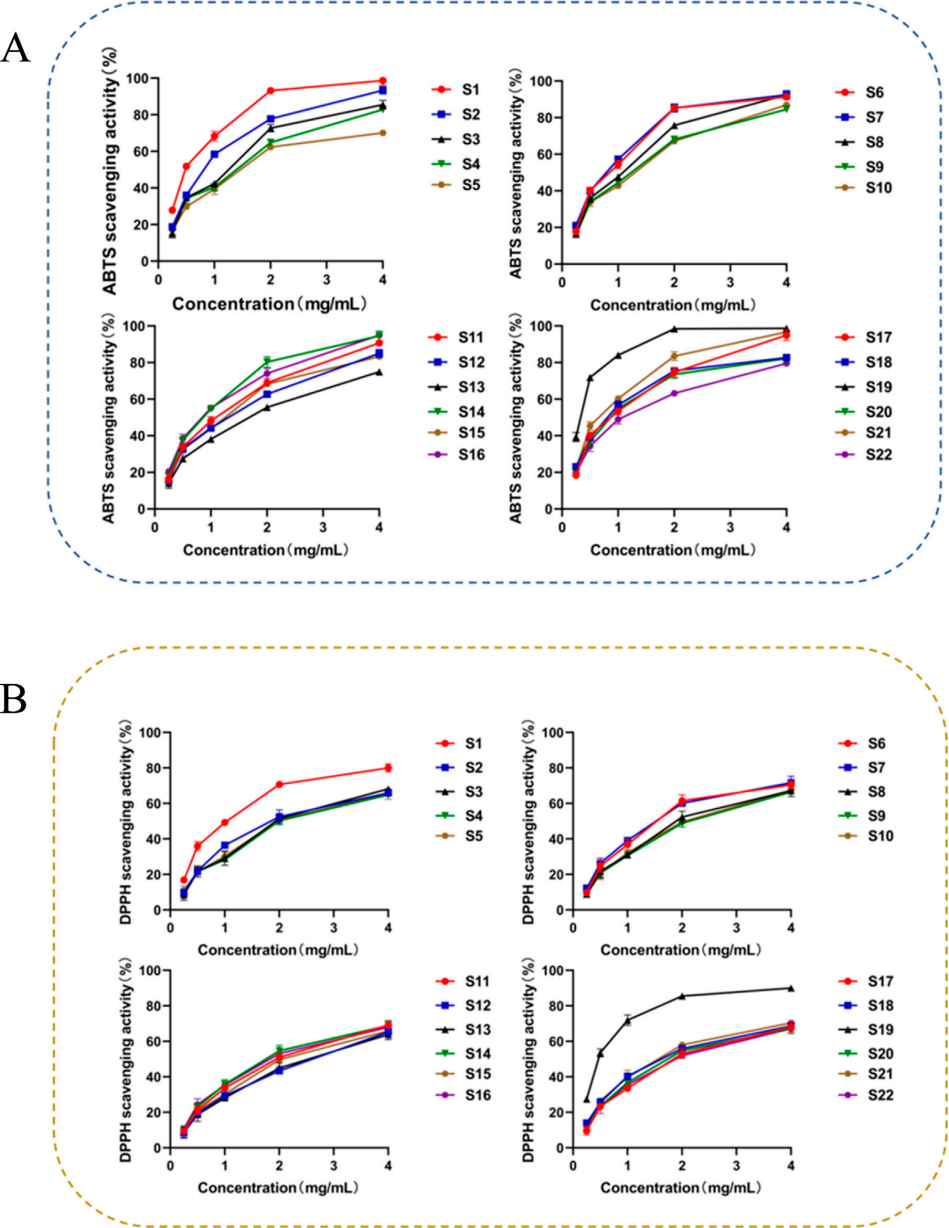
SZS free radical ability results show: **(A)** ABTS scavenging ability, **(B)** DPPH scavenging ability.

The IC_50_ value intuitively reflects a drug’s inhibitory or promotional effect on specific biological targets such as enzymes or receptors (Zheng et al., 2016). By comparing IC_50_ values across different drugs, their relative activity strengths on the same target can be assessed, which is crucial for identifying potent drugs (Abdin et al., 2019). The ABTS and DPPH free radical scavenging abilities and IC_50_ values for the 22 SZS sample solutions are detailed in Figure 4 and Supplementary Table S4. Concentrations are listed from left to right as 0.25 mg/mL, 0.5 mg/mL, 1 mg/mL, 2 mg/mL, and 4 mg/mL. Results indicated that SZS samples at varying concentrations exhibited antioxidant activity. Within a specific concentration range, activity strength increased with higher concentrations. A lower IC_50_ value indicates stronger activity. The IC_50_ values for ABTS and DPPH free radical scavenging indicated that samples S1 and S19 exhibited the best free radical scavenging effects, while samples S12 and S13 demonstrated relatively poor effects. There were certain variations in the ABTS and DPPH free radical scavenging abilities among the 22 SZS sample solutions. No significant differences in activity were observed among different regions, which might be associated with the contents of phenolic substances in different regions obtained previously. In addition to the differences in total phenolic content, the activities manifested by specific phenolic substances also varied, which might be the primary cause for the differences in activity.

### 3.7 Partial least squares regression

**FIGURE 5:**
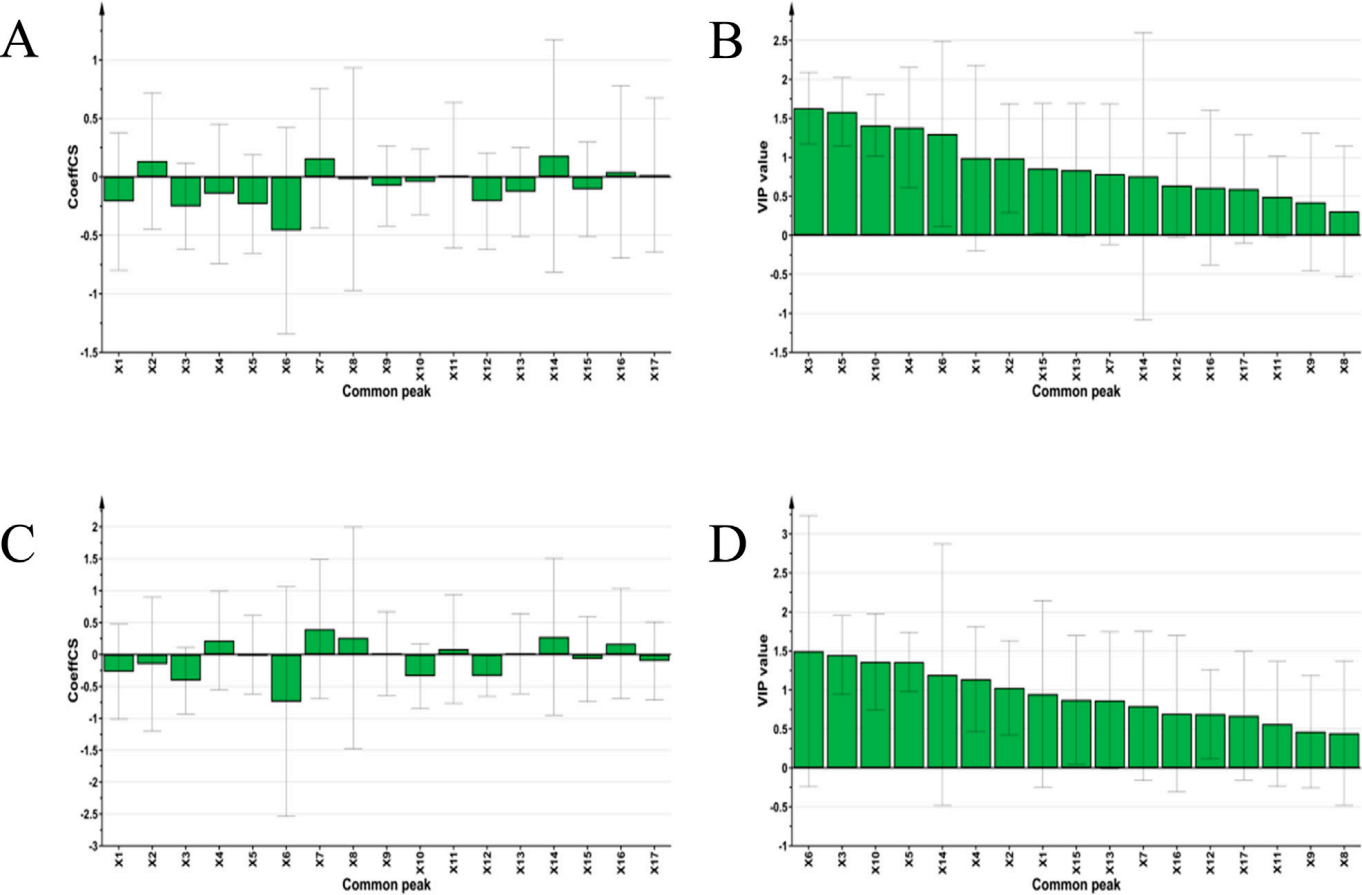
PLSR analysis of 22 SZS samples: **(A)** Regression coefficient diagram of common peak and ABTS scavenging ability, **(B)** VIP value diagram of common peak and ABTS scavenging ability, **(C)** Regression coefficient diagram of common peak and DPPH scavenging ability, **(D)** VIP value diagram of common peak and DPPH scavenging ability.

Using the peak areas of the common peaks from each SZS sample solution as independent variables (X), and the IC_50_ values of DPPH and ABTS free radical scavenging abilities as dependent variables (Y), these data were imported into SIMCA 14.1 software for PLSR discriminant analysis to explore the correlations between the common peaks and free radical scavenging abilities. By calculating the regression coefficients and the importance projection method (VIP values) for each variable, a coefficient greater than 0 signifies a positive correlation, while less than 0 indicates a negative correlation (Zhu et al., 2016). A higher VIP value indicates a greater contribution rate. Typically, VIP values greater than 1 are considered significant. Results indicated that, aside from X2, X7, X11, X14, X16, and X17 in the SZS fingerprint spectra, all other variables were negatively correlated with the ABTS free radical scavenging IC_50_ value (Figure 5A). Notably, peaks X6, X3, X5, X1, and X12 had the most significant inverse effect on the ABTS clearance IC_50_ value, indicating a stronger promotional effect on ABTS clearance. Considering VIP values greater than 1 as significant, the contribution to ABTS clearance ability, from greatest to least, is as follows: peaks X3, X5, X10, X4, X6, X1, and X2 (Figure 5B). Common peaks X6, X3, X10, X12, X1, and X2 were negatively correlated with the DPPH clearance IC_50_, suggesting a beneficial promotional effect on DPPH clearance (Figure 5C). Similarly, using VIP values greater than 1 as the benchmark, the contributions to DPPH clearance ability, from greatest to least: X6, X3, X10, X15, X14, X4, and X2 (Figure 5D).

### 3.8 Gray relational analysis

Grey relational analysis (GRA), frequently used to compare similarities and differences among various factors, effectively assesses the degree of correlation between multiple factors (Li et al., 2013). A higher relational degree indicates a stronger correlation between the two factors. A relational degree value above 0.6 is considered indicative of a correlation between the factors. Using the IC_50_ values of ABTS and DPPH free radical scavenging for the 22 SZS sample solutions as the reference sequence (parent sequence), and the peak areas of the common peaks from their fingerprint spectra as the comparison sequence (subsequence), GRA was performed to calculate and rank the relational degrees. Results are detailed in Table 1 The relational degrees between the 17 common peaks and the IC_50_ values for free radical scavenging were all above 0.6, indicating a correlation between each common peak and free radical scavenging ability. This also suggests that the antioxidant activity of SZS sample solutions results from the combined action of various phenolic compounds.

**TABLE 1:** Correlation between common peaks of SZS and antioxidant activity.

Serial number	DPPH scavenging activity		ABTS scavenging activity	
	Common peak	Relational degree	Common peak	Relational degree
1	X2	0.850	X2	0.825
2	X9	0.838	X14	0.804
3	X11	0.829	X11	0.803
4	X8	0.814	X15	0.794
5	X15	0.813	X9	0.789
6	X14	0.813	X8	0.783
7	X17	0.802	X16	0.780
8	X16	0.801	X13	0.780
9	X13	0.797	X1	0.774
10	X7	0.787	X7	0.769
11	X4	0.775	X4	0.754
12	X1	0.771	X17	0.735
13	X3	0.759	X3	0.734
14	X12	0.737	X12	0.714
15	X5	0.733	X5	0.710
16	X10	0.708	X6	0.688
17	X6	0.671	X10	0.656

### 3.9 Pearson correlation analysis

**FIGURE 6:**
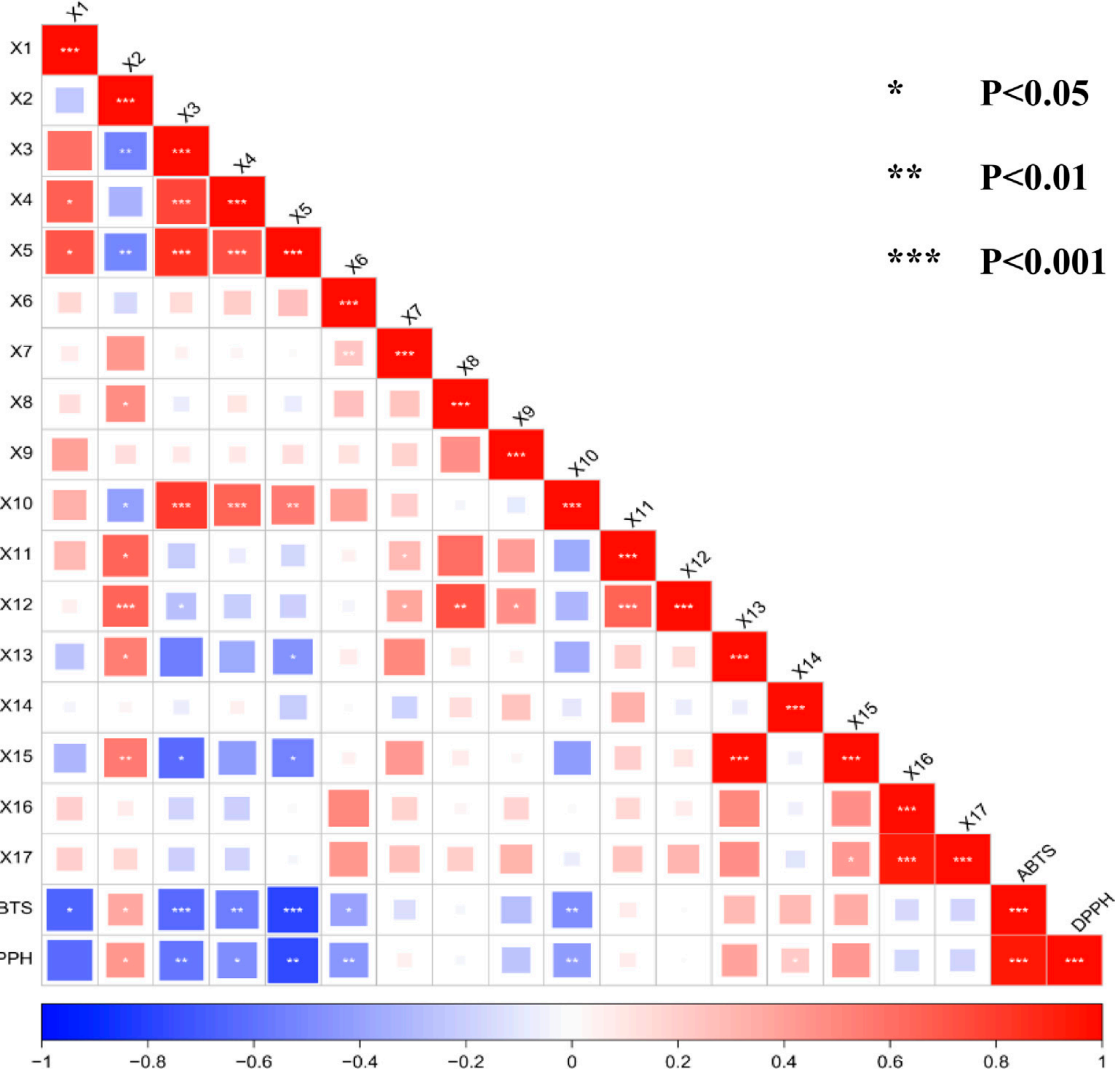
Pearson correlation degree between common peak and antioxidant activity of SZS.

Pearson correlation analysis is often utilized to assess the degree of correlation between two variables and to determine the direction of positive or negative correlations. A correlation coefficient (r) between −1 and 0 indicates a negative correlation, while 0 < r ≤ 1 indicates a positive correlation. Using the peak areas of the 17 common peaks from the SZS fingerprint spectra as variables, the correlation with the DPPH free radical scavenging rate and the ABTS free radical scavenging IC_50_ value was investigated. Analyzing the results shown in Figure 6, the peaks X1, X2, and X6 showed significant correlation with ABTS radical scavenging (P< 0.05), while peaks X4 and X10 exhibited extremely significant correlation with ABTS radical scavenging (P< 0.01). Additionally, peaks X3 and X5 had the strongest correlation with ABTS radical scavenging (P< 0.001). Similarly, we observed that peaks X2 and X4 were significantly correlated with DPPH radical scavenging (P< 0.05), and peaks X3, X5, X6, and X10 showed extremely significant correlation with DPPH radical scavenging (P< 0.01). Except for peak X2, which expressed a positive correlation as a light red color, all the aforementioned peaks were negatively correlated. It is worth noting that a lower IC_50_ indicates stronger activity. Therefore, the peaks described above with strong correlations promote the scavenging of ABTS and DPPH radicals, except for peak X2.

### 3.10 Discussion

In this study, using 22 SZS samples as the basis, chromatographic conditions including detection wavelength, column temperature, mobile phase composition, and gradient elution program were optimized. By comprehensively examining peak appearance time, peak shape, and response value, a total of 17 common chromatographic peaks were identified, and the HPLC fingerprint spectra for the phenolic compounds of the 22 SZS samples were successfully established. Comparison with the mixed reference sample chromatogram allowed for the identification of four phenolic compounds: gallic acid, catechin, spinosin, and scutellarin. Concurrently, quantitative analysis was performed using the external standard method.

Spinosin, a marker compound for SZS (Liao et al., 2012), plays a pivotal role in Ziziphus jujuba var. spinosa. Medicinally, it exhibits sedative and tranquilizing effects. Its mechanism of action involves affecting neurotransmitters (Xu et al., 2019), and it also possesses antioxidant and anti-inflammatory properties, as well as neuroprotective functions (Ge et al., 2022). Regarding clinical application prospects, it is a significant target for the development of Chinese medicinal formulations. SZS-based Chinese medicine preparations are already in use for treating insomnia and other symptoms, and can be combined with other drugs to potentiate their therapeutic effects (Li et al., 2006). Thus, the qualitative and quantitative analysis of spinosin in SZS holds significant importance. Catechins, gallic acid, and scutellarin also exhibit excellent performance in terms of antioxidant activity. Relevant studies have demonstrated the antioxidant activity conferred by oxygen-related functional groups and conjugated systems (Ramanan et al., 2016). Catechins, due to their unique structure, which includes internal conjugated systems and a multitude of hydroxyl groups, support their strong antioxidant activity (Sudarshan et al., 2017). In the study by Tanaka et al., it was found that knocking out Nrf2 significantly reduced the expression of antioxidant enzymes NQO-1, HO-1, CAT, and GCLM induced by gallic acid, suggesting that gallic acid upregulates Nrf2-targeted antioxidant genes, thereby enhancing the activity of antioxidant enzymes. Research by Pandurangan et al., indicates that gallic acid may regulate the cellular redox balance by activating the Keap1-Nrf2 pathway. Similarly, the phenolic hydroxyl groups and conjugated systems present in scutellarin contribute to the scavenging of free radical ions.

Crop rotation is considered one of the key strategies affecting plant cultivation and growth and development (Zuo et al., 2015). It can reduce the incidence of diseases by improving soil quality, increasing the number and abundance of beneficial microorganisms, and changing the structure of the soil community, thereby indirectly affecting plant growth and yield (Wang et al., 2010). Another study has shown that due to different preceding crops, the soil composition will be improved to different degrees, which will also directly affect crop quality (Larkin et al., 2015). From the subsequent analysis, it can be seen that the 22 SZS samples were divided into different categories, and there was an obvious correlation with the planting area. This may be because the preceding crops in different areas are different, and the soil environment has changed to different degrees, so the quality of SZS has shown certain differences. Combining the spectrum-effect relationship, it can be seen that different phenolic compounds have different promoting effects on the antioxidant activity of SZS. The common peaks X2, X3, X5, X6, X10 have the best comprehensive performance and play a dominant role in SZS. The final result also shows that the antioxidant activity of SZS is the result of the joint action of all its internal phenolic compounds. It is noteworthy that the spectrum-effect relationship is mostly used for the quality evaluation of the same plant in different regions and the screening of potential active substances in previous studies. For example, Shi et al. (2023) established the anti-inflammatory and analgesic spectrum-effect relationship of Chloranthus spicatus, constructed a quality assessment system, and screened out possible anti-inflammatory and analgesic substances. Nijat et al. (2021) used four chemometric methods to construct the spectrum-effect relationship between the UPLC fingerprint and biological activity of Rosa, providing a basis for its quality assessment and quality control. In this study, based on the regions with different preceding crops, the spectrum-effect relationship of SZS in the same region was constructed. At the same time, the correlation between the two was explored through chemical pattern recognition, providing a new idea for the further development of SZS in the future.

## 4 Conclusion

This study established the fingerprint spectra of phenolic compounds in SZS from various preceding cropping areas, constructed the spectrum-effect relationship with antioxidant activity, and identified 5 main influencing characteristic peaks, providing a foundation for future research and development of SZS-derived products.

## Data Availability

The original contributions presented in the study are included in the article/Supplementary Material, further inquiries can be directed to the corresponding authors.
