# Supplementary material for: Establishment of fingerprint of phenolic compounds in Semen Ziziphi Spinosae and study on the spectrum-effect relationship based on different preceding cropping areas
Source: Front Chem. 2025 Jan 3;12:1520586. doi: 10.3389/fchem.2024.1520586 (PMC11739076; doi:10.3389/fchem.2024.1520586)
Supplement: Supplementary file 1 [file Table1.docx]

**Supplementary Material**

TABLE 1 Regression equation and R^2^ of 4 phenolic compounds.

| Analytical object | Regression equation | Range (μg/mL） | R^2^ |
| --- | --- | --- | --- |
| Gallic acid | y = 1974.59x−2677.41 | 2.13~106.67 | 0.9999 |
| Catechin | y = 561.61x−121.83 | 1.60~80.00 | 0.9997 |
| Spinosin | y = 3235.26x−1453.18 | 1.64~150.00 | 0.9999 |
| Scutellarin | y = 2156.71x+10009.51 | 0.80~200.00 | 0.9999 |

TABLE 2 Contents of 4 phenolic compounds in 22 samples.

| Sample | Gallic acid （mg/g） | Catechin （mg/g） | Spinosin（mg/g） | Scutellarin （mg/g） | Total phenols （mg/g） |
| --- | --- | --- | --- | --- | --- |
| S1 | 1.47±0.04 | 0.94±0.05 | 2.89±0.29 | 2.40±0.17 | 17.13±0.41 |
| S2 | 1.14±0.07 | 0.75±0.03 | 4.75±0.33 | 2.52±0.36 | 20.57±1.68 |
| S3 | 0.47±0.03 | 2.21±0.12 | 6.42±0.74 | 3.64±0.49 | 16.63±0.53 |
| S4 | 0.75±0.06 | 1.40±0.08 | 10.09±0.08 | 5.08±0.08 | 21.44±1.26 |
| S5 | 0.95±0.11 | 1.42±0.19 | 6.74±0.20 | 6.04±0.69 | 14.42±1.05 |
| S6 | 1.24±0.16 | 2.32±0.18 | 6.88±0.16 | 6.20±0.81 | 20.36±0.32 |
| S7 | 0.97±0.14 | 2.45±0.35 | 7.24±0.43 | 4.02±0.58 | 18.90±1.80 |
| S8 | 1.09±0.03 | 0.75±0.03 | 6.79±0.16 | 5.78±0.39 | 19.61±1.21 |
| S9 | 0.91±0.12 | 0.82±0.09 | 7.92±0.89 | 4.75±0.32 | 18.67±0.54 |
| S10 | 0.96±0.02 | 0.83±0.11 | 6.88±0.29 | 4.68±0.31 | 16.29±0.21 |
| S11 | 0.65±0.08 | 0.64±0.01 | 7.32±0.76 | 4.16±0.39 | 15.93±0.58 |
| S12 | 1.05±0.08 | 1.26±0.13 | 10.31±0.41 | 5.11±0.73 | 20.42±2.64 |
| S13 | 0.69±0.10 | 0.67±0.02 | 6.72±0.81 | 3.42±0.20 | 11.63±1.61 |
| S14 | 1.26±0.05 | 1.22±0.01 | 6.56±0.95 | 4.66±0.69 | 18.95±0.41 |
| S15 | 0.68±0.08 | 1.16±0.14 | 4.97±0.56 | 1.94±0.22 | 14.35±1.45 |
| S16 | 1.33±0.05 | 3.53±0.29 | 7.22±0.49 | 8.63±1.00 | 23.89±0.48 |
| S17 | 0.90±0.06 | 0.82±0.04 | 6.76±0.90 | 4.31±0.15 | 17.99±1.42 |
| S18 | 0.96±0.09 | 2.38±0.17 | 4.10±0.53 | 4.01±0.56 | 14.79±0.69 |
| S19 | 1.11±0.16 | 3.98±0.04 | 6.52±0.37 | 4.20±0.56 | 21.63±2.68 |
| S20 | 2.33±0.08 | 0.64±0.09 | 6.71±0.75 | 6.45±0.15 | 20.39±0.45 |
| S21 | 1.13±0.11 | 3.48±0.28 | 2.75±0.25 | 3.79±0.30 | 17.81±1.27 |
| S22 | 0.97±0.09 | 1.41±0.21 | 4.15±0.24 | 3.02±0.28 | 16.97±1.68 |

TABLE 3 Similarity analysis of 22 batches of SZS.

| Sample | Similarity | Sample | Similarity |
| --- | --- | --- | --- |
| S1 | 0.832 | S12 | 0.940 |
| S2 | 0.886 | S13 | 0.922 |
| S3 | 0.983 | S14 | 0.987 |
| S4 | 0.980 | S15 | 0.977 |
| S5 | 0.944 | S16 | 0.954 |
| S6 | 0.993 | S17 | 0.992 |
| S7 | 0.991 | S18 | 0.974 |
| S8 | 0.991 | S19 | 0.985 |
| S9 | 0.977 | S20 | 0.987 |
| S10 | 0.994 | S21 | 0.886 |
| S11 | 0.972 | S22 | 0.944 |

TABLE 4 IC_50_ value of free radical scavenging ability

| Sample | ABTS scavenging activity IC_50_  （mg/mL） | DPPH scavenging activity IC_50_  （mg/mL） | Sample | ABTS scavenging activity IC_50_  （mg/mL） | DPPH scavenging activity IC_50_  （mg/mL） |
| --- | --- | --- | --- | --- | --- |
| S1 | 0.48 | 0.96 | S12 | 1.14 | 2.33 |
| S2 | 0.75 | 1.88 | S13 | 1.48 | 2.37 |
| S3 | 0.99 | 1.98 | S14 | 0.76 | 1.75 |
| S4 | 1.14 | 2.13 | S15 | 1.06 | 2.09 |
| S5 | 1.29 | 2.06 | S16 | 0.74 | 1.80 |
| S6 | 0.73 | 1.53 | S17 | 0.78 | 1.84 |
| S7 | 0.70 | 1.47 | S18 | 0.77 | 1.60 |
| S8 | 0.92 | 1.95 | S19 | 0.32 | 0.49 |
| S9 | 1.05 | 2.09 | S20 | 0.82 | 1.76 |
| S10 | 0.96 | 2.01 | S21 | 0.62 | 1.53 |
| S11 | 0.95 | 1.88 | S22 | 0.99 | 1.86 |
